# Stimuli Responsive, Programmable DNA Nanodevices for Biomedical Applications

**DOI:** 10.3389/fchem.2021.704234

**Published:** 2021-06-30

**Authors:** Udisha Singh, Vinod Morya, Bhaskar Datta, Chinmay Ghoroi, Dhiraj Bhatia

**Affiliations:** ^1^ Biological Engineering Discipline, Indian Institute of Technology Gandhinagar, Palaj, India; ^2^ Center for Biomedical Engineering, Indian Institute of Technology Gandhinagar, Palaj, India; ^3^ Chemical Engineering Discipline, Indian Institute of Technology Gandhinagar, Palaj, India

**Keywords:** DNA nanotechnology, stimulus responsive devices, biomedical applications, biosensing, therapeutics

## Abstract

Of the multiple areas of applications of DNA nanotechnology, stimuli-responsive nanodevices have emerged as an elite branch of research owing to the advantages of molecular programmability of DNA structures and stimuli-responsiveness of motifs and DNA itself. These classes of devices present multiples areas to explore for basic and applied science using dynamic DNA nanotechnology. Herein, we take the stake in the recent progress of this fast-growing sub-area of DNA nanotechnology. We discuss different stimuli, motifs, scaffolds, and mechanisms of stimuli-responsive behaviours of DNA nanodevices with appropriate examples. Similarly, we present a multitude of biological applications that have been explored using DNA nanodevices, such as biosensing, *in vivo* pH-mapping, drug delivery, and therapy. We conclude by discussing the challenges and opportunities as well as future prospects of this emerging research area within DNA nanotechnology.

## Introduction

There has been expanding attempt in the advancement of stimuli-responsive nanomaterials with the expectation that they can be formed into powerful diagnostic vehicles that can sense and deliver at the targeted or disease site *in vivo* (Stephanopoulos et al., 2013; Zhu et al., 2013; Bai et al., 2016; Rogers et al., 2016; Wang et al., 2016; Zhou et al., 2017; Wang et al., 2017a; Sawada and Serizawa, 2018; Sugimoto et al., 2019). Biomolecules already have encoded structural and functional information in them are nanoscale materials that can be modified and used to make human-made building blocks to form stimuli-responsive nanostructures. DNA (Rogers et al., 2016), protein (Bai et al., 2016), enzymes (Wang et al., 2016; Zhou et al., 2017), viruses (Sawada and Serizawa, 2018; Sugimoto et al., 2019) and others are potential nanomaterials for controllable self-assembly structure. The ordered system, adjustable functional groups, and unique properties at the molecular level allow these biological nanomaterials to be used in material science, tissue engineering, biosensors, biomedical engineering, and nanotechnology (Stephanopoulos et al., 2013; Zhu et al., 2013; Mauro et al., 2014; Wang et al., 2017a). The critical challenge is to control the self-assembly of biomolecules. Managing molecule-molecule interactions (such as hydrogen bonding, electrostatic interactions, DNA/RNA hybridization) or applying external stimulations (such as pH, temperature etc.) can solve this challenge. These biomolecular self-assembled nanomaterials’ applications can be improved by adding functional biocompatible nanoparticles like quantum dots, graphene, carbon tubes, and polymers. This assembly helps make hybrid nanomaterials that are more superior in terms of biomedical application than the individual nanomaterial. For example, Zao et al. synthesized photothermal peptide-porphyrin self-assembly based nanodots for anti-cancer therapy. These nanodots are biocompatible and suitable photothermal agents for cancer cell ablation (Zou et al., 2017). In Ravoo’s group, they constructed hydrogel of small molecular weight peptide (Nap GFYE) containing iron oxide paramagnetic nanoparticles. The hybrid hydrogel can quickly transform into gel to sol transition on the application of the external magnetic field. Such stimuli-responsive hydrogel shows significant potential for on-demand drug release applications (Nowak et al., 2021).

Recent discoveries in the field of DNA nanotechnology brings close attention to DNA self-assembly in several disciplines. DNA self-assembly can arrange heteroelements in a manageable fashion. It was Seeman’s idea that biomolecules like protein can be organized and oriented the same as DNA lattices. The ordered structure of proteins obtained, just like natural crystals, allow their study with X-ray crystallography (Seeman, 1982). These engineered frameworks use a grouping of endogenous or exogenous stimuli to initiate an assortment of reactions that can encourage targeted drug delivery. A set of endogenous stimuli is equipped for prompting changes in nanomaterial structure and functionality, vast numbers of which show changing articulation designs inside specific cell organelles or in unhealthy tissue (Rapoport, 2007; Ganta et al., 2008; De La Rica et al., 2012; Fleige et al., 2012). These improvements incorporate proteins (Verma et al., 2018), nucleic acids (Rosi et al., 2006), peptides (Feyzizarnagh et al., 2016), small particles (Decuzzi et al., 2017), electron transport reaction (Forsyth et al., 2017), osmotic pressure (Chien and Lin, 2002), viscosity, and neighboring environmental components, for example, pH (Foss et al., 2004), temperature (Nakayama et al., 2006), or redox state. Notably, while multiple frameworks prefer response towards normally emergent endogenous stimuli, more effort is focused on methods depending on exogenous stimuli. For example, ultrasound (Unger et al., 2001), electromagnetism (Xiao et al., 2012), temperature (Lee et al., 2021), and light (Wang et al., 2018) can be applied straightforwardly to the targeted tissue to control localization or cargo release (Rapoport, 2007; Ganta et al., 2008; De La Rica et al., 2012; Fleige et al., 2012). Evan et al. use ultrasound waves in localized delivery of DNA encapsulated in microbubble for gene therapy (Unger et al., 2001). When the microbubble exposed to ultrasound waves, cavitation occur locally, releasing DNA. Zeyu et al. developed DNA self-assembly targeted gold nanoparticles for cancer thermo-chemotherapy. They have designed 24 base pair DNA sequence for doxorubicin (Dox) loading. The DNA self-assembly is encapsulating Dox, conjugated onto gold nanorods. One of the DNA strands has NH_2_-terminated PEG-folic acid for targeted delivery of cargo to the cancerous cells. On providing, NIR radiation, the gold nanorods heat up, resulting in DNA denaturation and release of the drug (Xiao et al., 2012). Chen et al. in 2018 have synthesized photoresponsive nucleic acid-based carboxymethyl cellulose (CMC) based shape-memory hydrogels. The synthesized hybrid hydrogel mutually stabilized by photoisomerizable trans-azobenzene/β-cyclodextrin and DNA as cross-linker. The CMC acts as a backbone of the hydrogel. The presence of the carboxylic acid group on the CMC matrix provide sites for attachment of different functional groups. trans-azobenzene/β-cyclodextrin supramolecular complexes and duplex nucleic acid bridges bind to such sites. By treating the hydrogel to UV radiation, *trans*-azobenzene is converted to a cis form, which reduces its binding affinity to β-cyclodextrin, leading to low hydrogel stiffness (Wang et al., 2018). Such hydrogels can be used for localized and time-dependent delivery of drugs. Lee and coworkers have also developed smart DNA nanogels for stimuli-responsive release of cancer therapeutic drug. Gold nanoparticles were incorporated in these DNA nanogels for light-induced temperature increase. The temperature induced disassembly, therefore, shows precise control over the release of the loaded drug (Dox) (Lee et al., 2021).

Yu and coworkers developed DNA based shape memory DNA/acrylamide hydrogel strengthen by duplex nucleic acid and pH-responsive cytosine rich, I-motif. At pH 7.4, the I-motif bridges were separated, changing the hydrogel to a liquid-quasi shapeless state. The duplex DNA bridges are the permanent shape-memory element in the hydrogel. At pH five or Ag^˖^ ion, the quasi-liquid formless state of hydrogel reverse back to a normal stable condition (Yu et al., 2016). Temperature measurement at single-cell level is challenging and an important task to understand functional moieties in a complex system. Michael Famulok et al. developed thermal responsive DNA nanojoints. These DNA nanojoints are made of two interlocked double‐stranded DNA (dsDNA) rings. They can be switched from static state to mobile state at different temperature conditions without including any unique annealing process. The temperature response range of these nanojoints, which made up of DNA catenanes, can be tuned by changing the length and the sequence of hybridized part in the structure (Ma et al., 2020). As these stimuli may provide spatiotemporal authority for the activation of nanomaterials it is essential to coordinate the functional application with a suitable stimuli, to design responsive materials.

Deoxyribonucleic acid (DNA) has indicated extraordinary potential in the creation and development of nanostructures and devices. The double-stranded helical structure of DNA is key to its utilization in self-assembling applications. Using single-stranded overhangs, the double-stranded DNA can be engineered. The hybridization of two double-stranded DNA because of these overhangs leading to the formation of further self-assembly. DNA has advantages over others for forming devices and computational components, forming interconnects or as the device component itself. To start with, DNA is the molecule whose intermolecular interactions are the most promptly modified and dependably anticipated where G bonds C and A bonds T. Accordingly, the properties that make DNA hereditary material likewise make it a genuinely appropriate particle for programmed self-assembly. Second, DNA formed by different sequences can be obtained by solid support synthesis. DNA modification also takes place with biotin groups, and fluorescent markers presented new DNA applications in nanobiotechnology. Third, DNA can be modified using different enzymes that incorporate restriction endonucleases, exonucleases, and DNA ligases.

**FIGURE 1:**
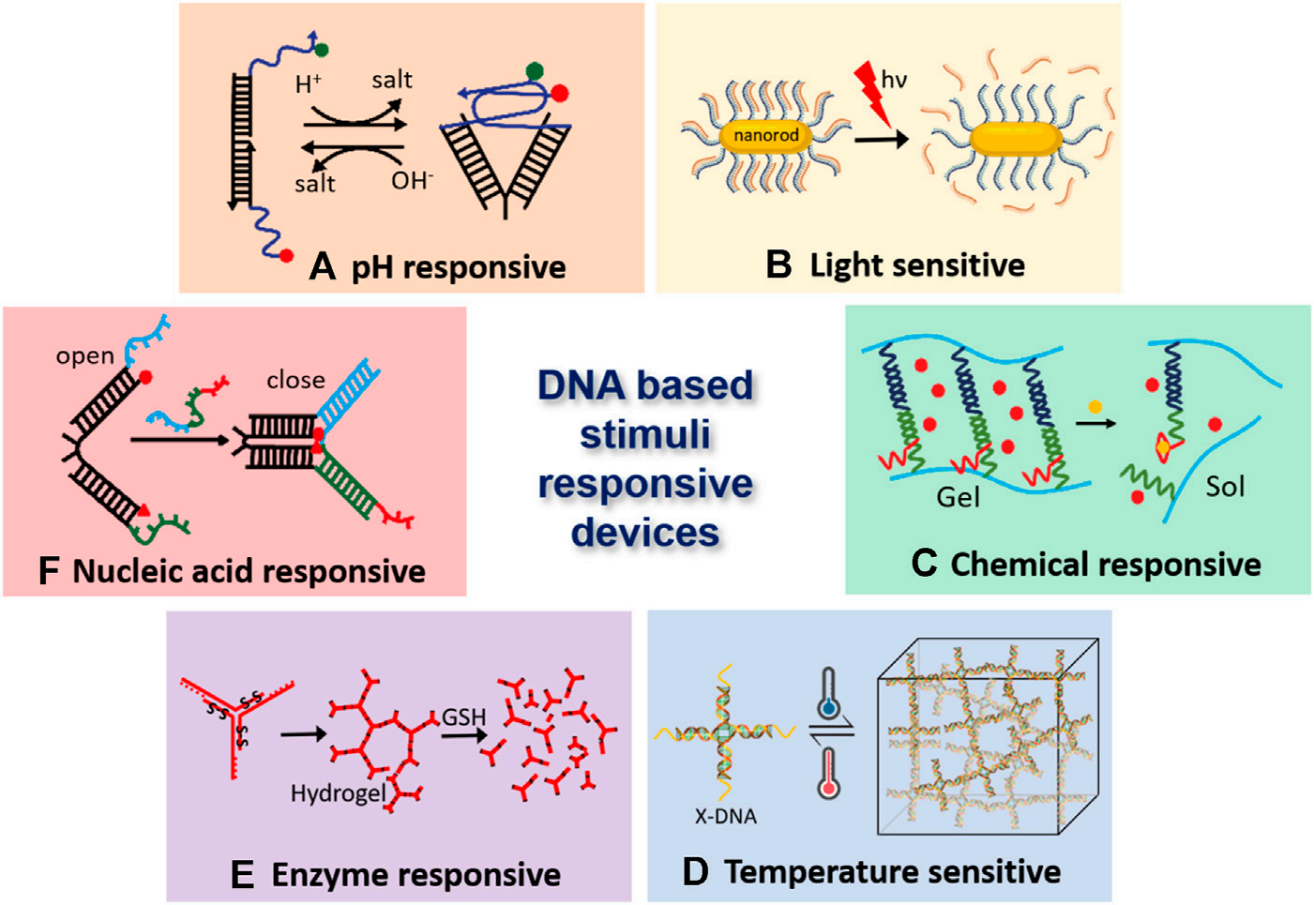
DNA-based stimuli-responsive nanodevices. **(A)**. DNA i-motif based pH responsive nanodevice (Surana et al., 2011), **(B)**. Light-responsive DNA-gold nano particles (Xiao et al., 2012), **(C)**. Hydrogel crosslinked with DNA aptamer, which enables it’s conversion from gel to sol and triggers the release of embedded fluorescent particles (Yang et al., 2008), **(D)**. Temperature sensitive DNA hydrogels, where GC content of the sticky ends decides the association-dissociation point (Xing et al., 2018), **(E)**. Hydrogel monomers having disulfide linkages breaks apart due to enzyme activity of glutathione (GSH) (Li et al., 2015), and **(F)**. Nucleic acid responsive nanotweezers act upon hybridization with target nucleic acid (Yurke et al., 2000).

According to the need, both single and double-stranded DNA are part of many devices, which can be used both as flexible and rigid molecular parts. A capable blend of these components passes on specific mechanical and chemical properties to the resultant devices. ssDNA can be used both as a flexible component and accessible molecular tags to which its complementary strand can easily bind. dsDNA is ordinarily utilized as inflexible structure blocks yet may likewise add to the devices’ chemical function by including chemical modifications and binding sites. Based on the principle of Holiday junction, 4 DNA strands could be self-assembled into rigid 4-way junction (Seeman, 2003). Double crossover (DX) tiles, triple-crossover (TX) tiles and paranemic-crossover (PX) tiles having excellent rigidity were used to create versatile DNA nanostructures, including both 2D and 3D architectures (Liu et al., 2004; Endo et al., 2005; Liu et al., 2008). The dsDNA is also used for the “mechanochemical” functioning of many devices. The thermodynamics and kinetics of formation of the double-stranded structure of DNA as well as the mechanical properties of both single and double-stranded DNA play a significant role not only in construction but also in the functioning of DNA-based nanodevices which can act as “smart programmable stimuli responsive materials for biological and biomedical applications” (Figure 1).

## Designing DNA Nanodevices for *In Vivo* Applications

**FIGURE 2:**
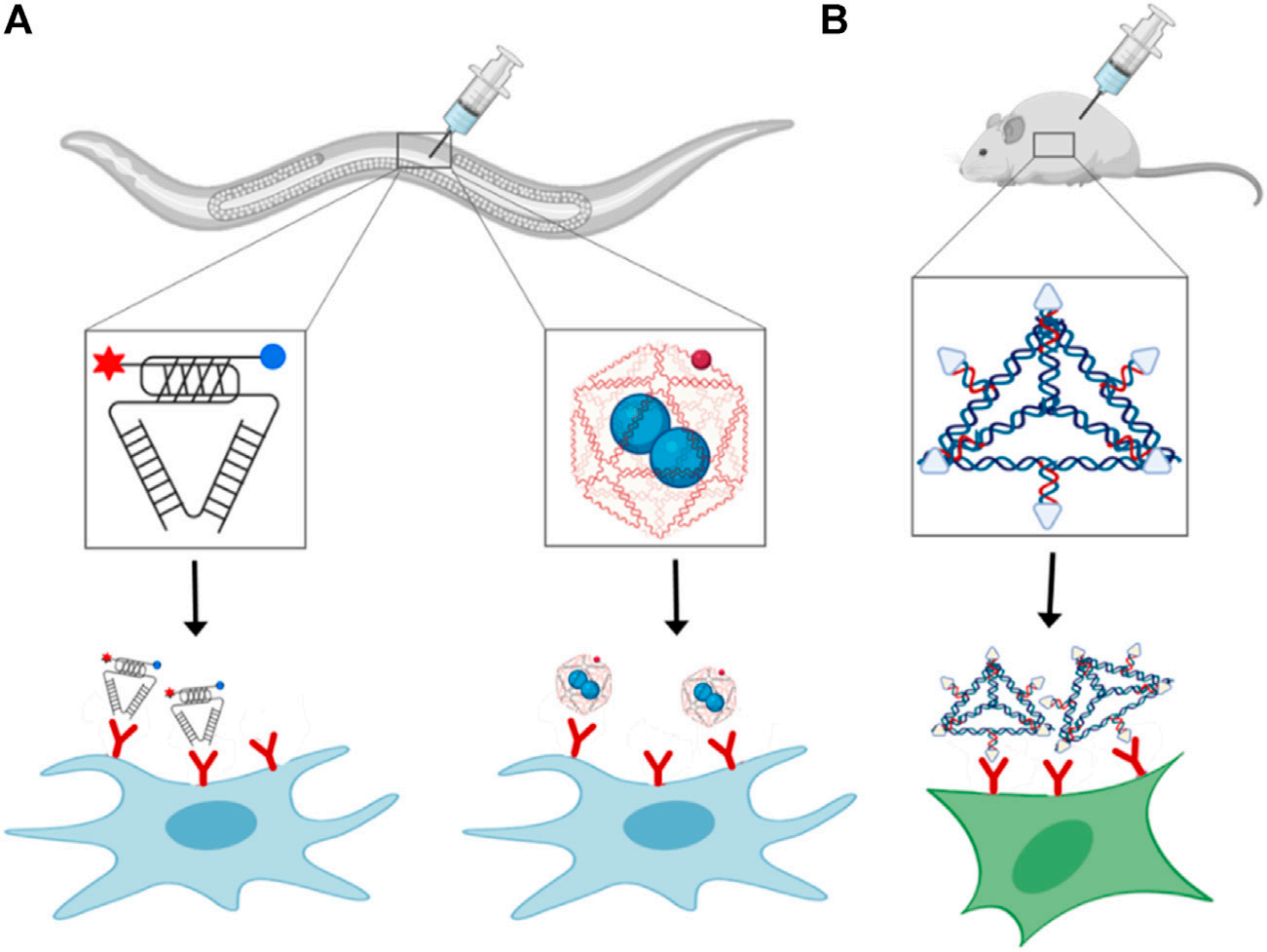
Receptor binding and delivery of DNA nanodevices: *In vivo* delivery of **(A)** i-motif based pH-responsive DNA nanodevice **(left)**, cargo loaded DNA icosahedron **(right)**, and **(B)** DNA tetrahedron functionalized with folate-moieties for siRNA delivery.

Although a few DNA based nanodevices have been applied to cells in culture, their application in multicellular life forms has barely arisen. The most crucial issue for *in vivo* applications of nucleic acid nanodevices at the cellular level is their stability and kinetics. The effective concentrations in the crowded cellular environment differ from those in the standard *in vitro* conditions where experiments are performed in well-mixed buffer systems. This leads to a very different volume and osmotic pressure effects, influencing the structure of DNA (Miyoshi and Sugimoto, 2008). For example, G-quadruplex structures in telomeres or three-way junctions (Muhuri et al., 2009) can be stabilized under molecular crowding conditions. A few designer DNA nanodevices have been used for biomedical applications like drug delivery and diagnostic probes in living systems (Krishnan and Bathe, 2012). Apart from the stability, the significant primary molecular barriers faced *in-vivo* are targeted delivery to the site of interest and toxicity towards the host organism. Currently, the predominant method of delivery of DNA nanodevices dependent on their injections to specific cell types. The first study of stimuli-responsive DNA nanodevices on a multicellular organism was done using a responsive DNA nanodevice, the I-switch. This I-switch was injected into *Caenorhabditis elegans*. The I-switch targeted specific scavenger cells that show anionic cell surface ligand-binding receptor (Figure 2A). Once it is internalized, the I-switch could probe endosomal maturation (Surana et al., 2011). Similarly, DNA icosahedron is used for drug delivery. to scavenger cells, in this case, both device stability and cargo functionality were preserved after delivery of drug (Bhatia et al., 2011; Surana et al., 2013) (Figure 2A).

DNA nanodevices have also been delivered intravenously in mammalian models. An overall designing rule for focusing on the nanodevices to the site of interest exploits the presence of specific ligands on the nanodevice that empowers its binding to a cell-specific endogenous receptor, prompting its cell take-up. Tetrahedron DNA nanoparticles can target cancerous cells by exploiting the overexpression of folate receptors on cancer cells. Tetrahedron nanodevices having folate moieties and siRNA are used for targeted delivery to xenograft tumor in nude mice (Figure 2B) (Lee et al., 2012a). After the internalization of the tetrahedron nanoparticles inside the cell, siRNA reduces the expression level of the target gene (Lee et al., 2012a). However, despite the targeting of DNA nanodevices to specific tissues, some nanoparticles are taken up by non-cancerous cells expressing folate receptors on their surface, making this strategy less specific (Antony, 1996). Out of various delivery modes, oral delivery is quite famous in animals, but not much work done so far in delivering DNA nanodevices through this delivery model. The main reason can be that DNA nanostructures are highly susceptible to acid-catalyzed digestion, nucleases and cannot easily pass through cell barriers. Apart from nucleic acids, other biomolecules such as proteins and peptides are primarily packed in nanoparticles, polymers, dendrimers, and micelles to prevent destruction from low intestinal pH and proteolysis (Longmire et al., 2008; Gupta et al., 2013). Intranasal delivery shows very promising results. There are rapid absorption and less metabolism rate of nanoparticles delivered through this method because of the high permeability and surface area of nasal endothelial cells. Also, this method of administration is non-invasive and user friendly (Ali et al., 2010). This method also provides access to the mammalian central nervous system, whose pathways are still not well understood (Dhuria et al., 2010). One such example is plasmids coated with polycationic lysine derivatives have been administered intranasally in rats leading to green fluorescent protein expression in the brain (Harmon et al., 2014). Other routes to deliver DNA nanodevices to specific tissues are direct injections to the target site. Most frequently used are chitosan-DNA nanoparticles injected into rat brain (Yurek et al., 2011), liver (Dai et al., 2006), eye (Farjo et al., 2006; Ding et al., 2009), and lungs (Ziady et al., 2003). Direct tissue infusions present great potential as they give high local concentrations and limit toxicity yet are intrusive and require exceptional mastery. Critically, despite the delivery course, DNA nanostructures will probably evoke an immune response that would be either utilized properly or moderately reduced depending on the functionality of the DNA nanostructure, like a vaccine or therapeutic cargos.

There are several cellular mechanisms to dispose of the extra DNA from the system to maintain homeostasis, so the efficiency and stability of DNA nanodevices need to match the applications for which they are being used. Stability in the organism’s circulatory system depends on many complex factors such as digestion from DNases, shape, size, cellular uptake, and removal of DNA nanodevices from circulation through the liver and kidney. For example, Shih and coworkers improve the stability of DNA nanostructures by coating them with PEGylated oligolysines which protect the DNA nanostructures against low salt concentration and inhances nuclease resistance up to 400 folds. They further increase the nuclease resistance up to more than 250 folds and incubation time more than 48 h in Dnase I solution by using glutaraldehyde, crosslinking PEGylated oligolysines. DNA nanostructures coated with cross-linked oligolysines are biocompatible, and their internalization is easy inside the cells compared to non-coated DNA nanostructures (Anastassacos et al., 2020). The size and shape of different nanoparticles like gold, silica, carbon, quantum dots, dendrimers, and liposomes affect their clearance time (Longmire et al., 2008) and cellular uptake (Doshi and Mitragotri, 2010) *in vivo* condition. Similar studies on DNA nanodevices are in progress. For example, Bhatia et al. studied the uptake, kinetics, and dynamics of DNA cages of different and found out that there is a pattern in uptake of DNA nanodevices with respect to the geometry of ligand and type pathway they are endocytosed (Hivare et al., 2021). Through the understanding of endocytic uptake and intracellular pathway of DNA nanodevices will help in designing targeted therapeutics. Furthermore, if the DNA nanostructures are partially dissociated, it will trigger a strong immune response, elevated toxicity and higher off-target delivery. Thus, both the stability of DNA nanostructure and cellular uptake pathway cumulatively defines their bioavailability.

## DNA Based Stimuli-Responsive Devices

Because of the incredible programmability of DNA molecules, both static DNA nanostructures and sensitive dynamic devices could be planned and built. In this manner, DNA nanotechnology is at times isolated into two subfields: primary DNA nanotechnology and dynamic DNA nanotechnology. The objective of DNA nanotechnology is to combine and modulate higher arranged DNA models, for example, one-dimensional (1D) nanotubes (Huang et al., 2019), two-dimensional (2D) arrays (He et al., 2005; Rothemund, 2006; Wei et al., 2012), and different limited or occasional three-dimensional (3D) structures (He et al., 2008; Zheng et al., 2009). Dynamic DNA nanotechnology means to manufacture different dynamic reconfigurable nanoscale devices, which function in a controllable way depending on using different chemical or physical stimuli. Indeed, stimuli-responsive DNA self-assembly joins the highlights of both primary and dynamic DNA nanotechnology. Stimuli-responsiveness of DNA nanostructure can be grouped into six categories. 1. The protonation of nucleobases presents some non-Watson-Crick base-pairing interactions in nucleic acids (e.g., Hoogsteen hydrogen bonding), which are profoundly sensitive to pH change. It permits the development of secondary DNA structures in somewhat acidic conditions, for example, triple-stranded helices (trio) and four-stranded intercalated motif (i-motif). This component has been used to plan pH-responsive frameworks. 2. Toehold-mediated strand displacement: Strand-displacement in DNA is fueled by the free energy of DNA hybridization, started by an overhanging region present in DNA called “toehold.” 3. DNA aptamer-target relation: DNA aptamer has high specificity and affinity toward its target, which can upgrade to responsive DNA self-assembly. 4. DNA chemical modification using stimuli responsive groups. The chemical molecules introduced into the system will strongly affect DNA self-assembly both by chemical or physical signal. Modification of DNA using azobenzene moieties helps in achieving the UV/Vis light-responsive switches. 5. Intervention through existing environmental molecules. The communications among DNA and molecules existing together with DNA, intervened by electrostatic, intercalative, and other different processes, could fill in as managing factors for DNA self-assembly. For instance, pH-responsive self-assembly and dismantling of DNA nanostructures have been accomplished through controlling the protonation and deprotonation of ethylenediamine in a buffer solution (Liu et al., 2017): 6. Base-stacking association: In base-stacking, a non-covalent attractive force exists between neighboring bases with a face-to-face arrangement, which has been exhibited to apply a huge impact on the self-assembly of DNA. For the most part, the initial three classes depend on explicit DNA arrangements, for example, DNA aptamer and i-motif. The last three classifications are sequence-independent DNA, without including specificity in sequence designs. It’s imperative that sometimes more than one trigger is used to accomplish far-off and more complex controls on dynamic DNA self-assembly. Stimuli-responsive DNA self-assembly has discovered extraordinary applications in different fields, for example, biosensing, drug conveyance, diagnostics, and nanorobotics. The hybridization chain reaction (HCR) has been generally used to design different sensors, including electrochemical and fluorescent sensors (Bi et al., 2017). Targeted drug delivery and treatment have been accomplished through a controlled opening of an origami nanorobot dependent on DNA aptamer-target interaction (Li et al., 2018).

## Key Examples of Different Stimuli Responsive DNA Nanodevices

### Motifs

**FIGURE 3:**
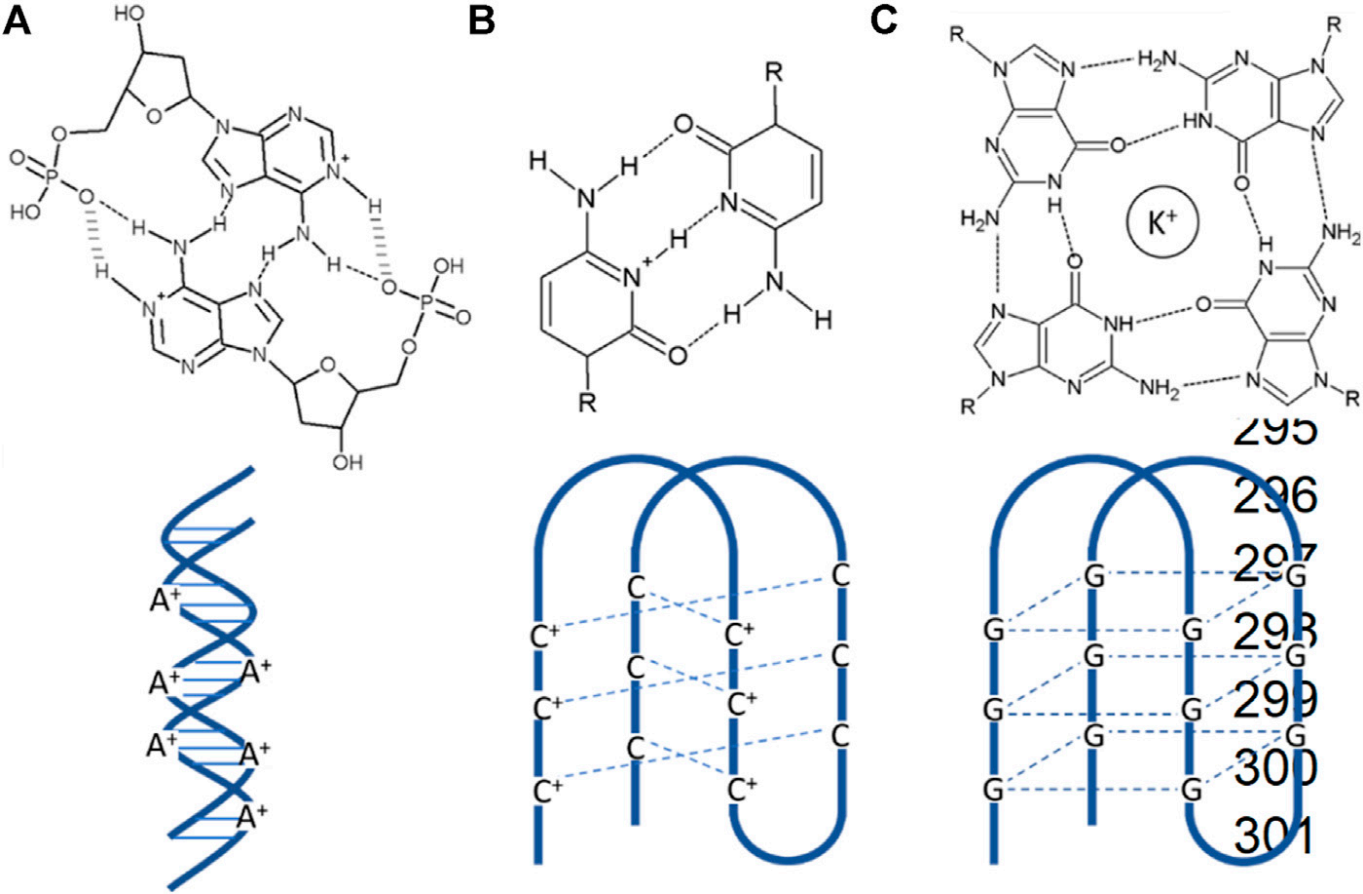
Different kinds of motifs **(A)** A-motifs are pH-induced AH^+^–H^+^A base-paired right-handed symmetric parallel-stranded duplex form of poly dA sequences with a highly reversible nature. **(B)** I-motifs are pH-induced intercalated C-rich DNA quadruplex with C-C^+^ base pairing. **(C)** G-quadruplexes are formed by guanine tetrads stacked in G-rich DNA or RNA sequences.

Homopolymer DNA synthesized artificially was used for studying base-stacking in DNA duplex structure as they were considered simplified models (Peters and Maher, 2010). At last, it was discovered that these artificially synthesized homopolymers formed diverse conformations, including non-Watson–Crick base pairing. A-motifs having a structure of parallel duplexes are formed by A-rich DNA (Figure 3A). C-rich DNA sequences include i-motifs or i-tetraplexes (Figure 3B). G-rich DNA sequences form G-quadruplexes (Figure 3C). G-quadruplexes are considered by many as a potential anti-cancer target. The incrimination of G-quadruplexes gives rise to several biological dysfunctions leading to selectively alternation of the integrity of cancer cells (Oganesian and Bryan, 2007). Specifically, the arrangement of G-quadruplex-DNA towards the end of telomeres has been accounted for not only to hinder the telomerase interconnection and activity but also severely hampering genomic stability by hindering the recognition ability of telomerase binding proteins to their targets (Kelland, 2007; De Cian et al., 2008). Many cytotoxic anti-cancer drugs such as Gemcitabine are explicitly delivered to cancerous cells using G-quadruplexes as vehicle (Park et al., 2018). The folding and unfolding of G-quadruplexes in response to environmental stimuli made them excellent signal transducer. Small metal ions such as K^+^, Cs^+^, etc., are the first line stimuli that controls the stability of G-quadruplex. That is why the G-quadruplex has been used as a sensing device for metal ion detection and other complex samples. The G-quadruplex are highly cation dependent, so they are quickly being used as fluorescent bio detector for metal ion detection such as potassium and copper (Nagatoishi et al., 2005; Kong et al., 2009; Qin et al., 2018). Small molecules like ATP and cocaine form the second category stimuli for G-quadruplex. Proteins, DNA, and other such biomolecules form the third category of the stimuli.

Octameric structures are formed by GU rich sequences where G tetrads and U tetrads are intercalated, which lead to the display of 8Us in an ordered spatial orientation (Nagatoishi et al., 2011). Petraplexes are created by iGuanine (iG) by narrowing down the angle of Watson-Crick base pairing and Hongsteen hydrogen bonding sites being displayed (Zhao et al., 2009). DNA repeats occurring naturally bear the cost of an asset of surprising structures. Numerous genomic sequences comprising of expandable repeats wind up shaping many unordinary motifs, such as quartets composed of (CGG)_n_ repeats, an imperfect hairpin structure formed of (CNG)_n_ repeats, slip-stranded DNA (Pearson and Sinden, 1996), and many more such type of structures.

### Aptamers

An aptamer is formed of nucleic acid sequences (DNA or RNA) having a length of 15–40 nucleotides or even longer and can bind specifically to a given molecular target. Nucleic acid (Mayer et al., 2011) forms a 3D structure when dispersed in solution. The shape attained by the aptamer decides its binding site to which the targeted molecule can easily bind. On the other hand, aptamers may be preorganized in a form and, through an induced-fit mechanism, bind to their target site. Aptamers can be created against any biological target in a test tube through a procedure called “systematic evolution of ligands by exponential enrichment” (SELEX) (Ellington and Szostak, 1992). These molecules can adopt extraordinary shapes based on the massive number of permutations possible in nucleic acid sequences. A considerable number of molecules can be targeted through these aptamers, such as small molecules, toxins, different classes of proteins, and even whole cells. Aptamers show exquisite specificity and bind to their target with high affinity (Kawazoe et al., 1997). The dissociation constant K_d_ of aptamers majorly shows nanomolar regime in case of proteins, and for small molecules, it goes to micromolar regime. Aptamers offer several advantages over antibodies because of their small size, as they are made up of a short length of nucleic acids. Aptamers can be easily synthesized *in vitro* as compared to antibodies leading to less variability in every batch. They can be easily labeled without disrupting target affinity and more excellent stability to sustain at high temperatures. Low molecular weight help aptamers to have excellent pharmacological properties like better target accessibility, short circulation time, and rapid clearance.

**FIGURE 4:**
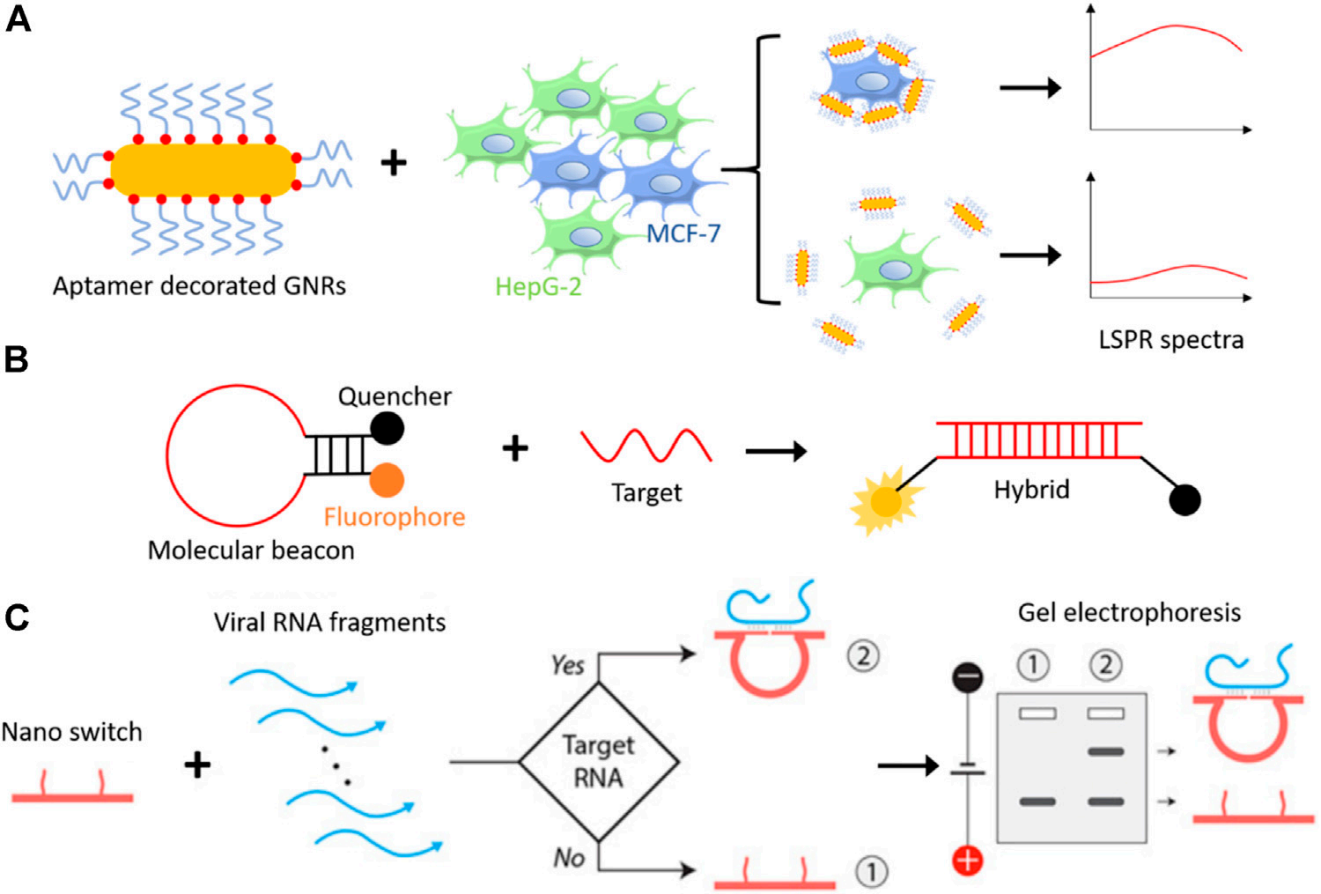
**(A)** Schematic representation of aptamer-GNRs based cancer detection system, where mucin-1 aptamer-decorated GNRs detect breast cancer cells (MCF-7) specifically among other cells and generate a higher signal on LSPR spectra (Li et al., 2016a). **(B)** Molecular beacons, a hairpin loop DNA structure with a fluorophore, and a quencher attached on each end remain in proximity until a target DNA/RNA molecule hybridizes with it. Hybridization opens the hairpin structure and fluorescence can be seen as read-out (Tyagi and Kramer, 1996). **(C)** Representation of DNA nanoswitch based detection of a target viral RNA (Zhou et al., 2020). DNA nanoswitch changes structural configuration from linear strand to a loop, when introduced to a complementary target DNA/RNA molecule, which results into slower mobility on gel electrophoresis. Reproduced with permission from ref Zhou et al. (2020). Copyright 2020, American Association for the Advancement of Science (AAAS).

Aptamers used for diagnostic purposes are conjugated with nanoparticles for enabling detection. Zhao et al. synthesized aptamer-modified silica fluorescent nanoparticles (FSNPs) by conjugating amine-labelled Sgc8 aptamer to carboxyl-modified FSNPs *via* amide coupling for the detection of leukemia cells (Tan et al., 2016). The sensitivity and selectivity of Sgc8-FSNPs accessed by flow cytometry and fluorescent spectrometry. Li et al. developed a simple biosensor for the detection of human breast carcinoma MCF-7 cells by functionalized aptamer on gold nanorods (GNRs). GNRs show unique optical properties with longitudinal plasmonic peaks, which can be controlled between visible to the near-infrared regions by changing the morphology, hence considered a potential candidate for therapy and imaging *in vivo*. The mucin-1 protein is one of the reported cancer biomarkers in human beings, expressed on cancer cells’ surfaces. Li and colleagues detected MUC-1 positive human breast carcinoma MCF-7 cells using specific interaction between MUC-1 and its aptamer, covalently conjugated to the surface of GNRs by Au-thiolate chemistry and scan the signals with unique localized surface plasmon resonance (LSPR) spectra (Li et al., 2016a) (Figure 4A). Recently, a one-pot fluorescence-based detection of the SARS-CoV-2 virus is introduced (Chandrasekaran et al., 2020; Woo et al., 2020). Which involves a promoter probe consists of an upstream hybridization sequence (UHS) to target the pathogenic RNA and a reporter probe consists of a downstream hybridization sequence (DHS) and a dye-binding aptamer template sequence. In this case, the target RNA acts as a trigger. When it correctly binds to the UHS and DHS, SplintR ligase connects the probes; subsequently, T7 RNA polymerase synthesize the aptamer sequence that attaches to the dye and gives fluorescence as a read-out.

The chemical modification of aptamers lead to a different variety of this oligonucleotide and particularly appealing for ligand-aptamer interactions in solution, called “molecular beacons” (Liu et al., 2009). Molecular beacon made up of original aptamer sequence extended with linker sequence forming the loop followed by the complementary series which helps in the formation of hairpin structure (Figure 4B). To each end of the hairpin structure, either fluorophore or quencher are attached; as the fluorophore is near the quencher in the closed state, there is no fluorescence signal. However, once the molecular beacon is attached to the ligand, as they are released to the target site, we start getting fluorescence signal because fluorophore and quencher are apart due to disruption of stem hybridization. The term “molecular beacon” used synonymously with “aptamer hairpin structure” or “aptamer switch probe” throughout the literature, so it should not be confused as separate nanodevices.

To this date, only a selected number of aptamers sequences targeted for small molecules to whole cells have been known so far. The limited use of aptamers is that automated aptamer selection procedures still require time and effort. As the selection procedure is conducted on-demand, there is still a tiny market for efficient chemicals (Keeney et al., 2009).

### DNA Nanoswitches

DNA nanoswitches work on a similar principle, where it binds to target DNA or RNA instead of a non-nucleic acid target. A DNA nanoswitch is designed to undergo a specific structural change when introduced to the target DNA or RNA sequence with a detection limit of picomolar range (Chandrasekaran et al., 2016). The prepared DNA strand switching from linear (“off” state) to loop (“on” state) and can be separated by electrophoresis without any amplification step. A programmable DNA nanoswitch-based low-cost viral RNA detection system has been realized recently to detect deadly viruses like Zika, Ebola, and SARS-CoV-2 (responsible for coronavirus disease 2019, COVID-19) virus, etc (Zhou et al., 2020). This method involves simple steps like sample collection, RNA isolation, RNA fragmentation (either enzymatic or non-enzymatic), annealing, and electrophoresis (Figure 4C). Unlike RT-PCR (reverse transcription-polymerase chain reaction) based detection methods, this may be used in low-resource areas at low cost.

### DNA Nanozymes

Many enzymes based on nucleic acid demonstrate metal ion-based catalytic activity. RNA molecules, also called Ribozymes showing catalytic activity, occur both naturally and artificially through SELEX (Wilson and Szostak, 1999). Equivalent to Ribozymes are DNAzymes (SilvermanDeoxyribozymes, 2009) which are synthesized through artificial method only. These molecules are engineered to folds into three-dimensional malleable structures, which offer catalytic centers and binding pockets. Most of these molecules are selected based on phosphoester transfer reaction and is extensively studied (Wilson and Szostak, 1999). The test tube synthesized DNAzymes can catalyze different chemical reactions such as Aldol reactions (Fusz et al., 2008), Diels-Alder reactions (Seelig and Jäschke, 1999), Michael reactions (Sengle et al., 2001), acylation (Murakami et al., 2003), and N-glycosidic bond formation (Unrau and Bartel, 1998). These functional nucleic acids found significant application in sensing, therapeutics, and targeted delivery. Different units from aptamers and DNAzymes, which play an important role in their functioning, are combined to give allosteric aptamers or “aptazymes.” In the case of proteins, allostery includes spatially separated catalytic sites that interact with each other through a change in their conformation when one of the effector molecules binds to one of the binding sites. The same principle is applied in the case of aptazymes. Such DNA nanostructures are of keen interest in the field of information processing, signal transduction, and also in biosensing (Burgstaller et al., 2002).

**FIGURE 5:**
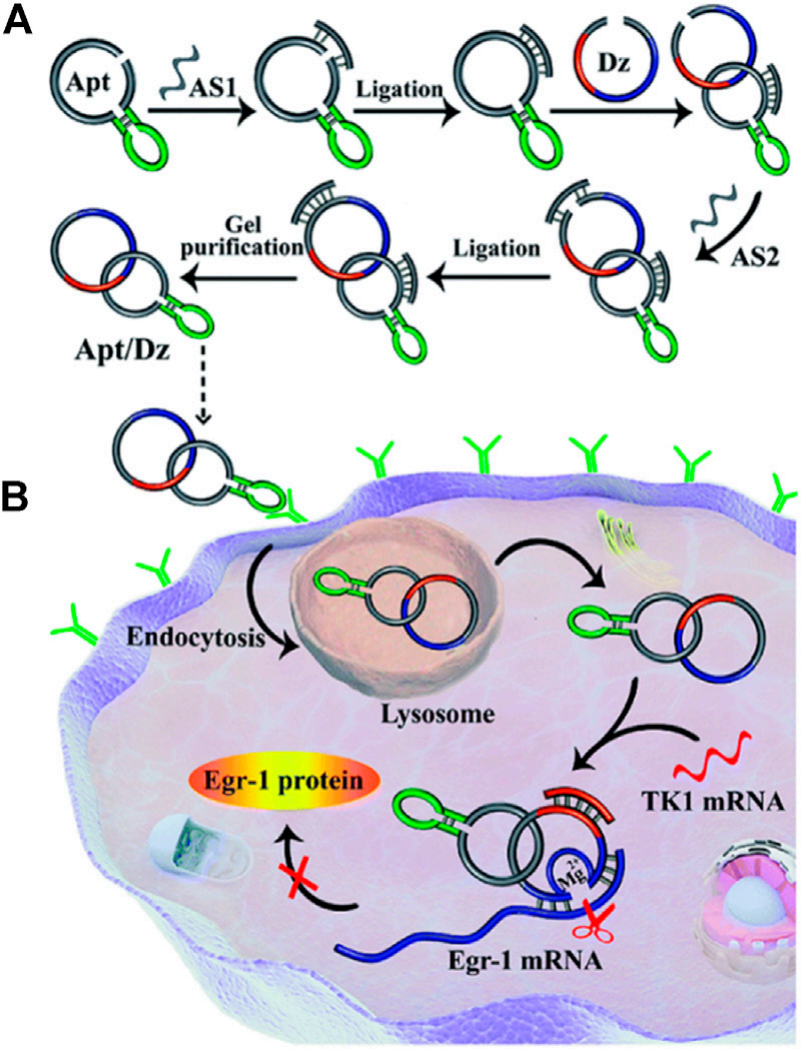
**(A)** Construction of the Apt/Dz constrained catenane nanostructure and **(B)** the representation of targeted delivery of the Apt/Dz nanostructure for gene silencing into cancer cells. Reproduced with permission from ref Li et al. (2020). Copyright 2020, The Royal Society of Chemistry (RSC).

Xia li and colleagues reported targeted delivery for highly specific gene silencing using stimuli-responsive aptamer/DNAzyme (Apt/Dz) catenane nanostructures. They synthesized thymidine kinase 1 (TK1) mRNA-responsive Apt/Dz (Figure 5A). The 10-23 DNAzymes were selected based on their RNA cleavage and used to aim at the early growth response-1 (Egr-1) gene. Egr-1 is vital for both tumor angiogenesis, growth as well as neovascularization (Fahmy et al., 2003; Mitchell et al., 2004). MCF-7 tumor cells were used inside the cells with elevated TK1 mRNA concentration (Ding et al., 2016; Zhong et al., 2018), and mucin1 (MUC1) proteins shown by the cancerous McF-7 cells were used for targeted delivery of Apt/Dz nanostructure. The Apt/Dz nanostructures, once incubated with MCF-7 cells, enter the cell through the endocytic pathway by binding to the MUC-1 proteins expressed on the surface of the cell membrane. TK1 mRNA hybridizes with these nanostructures, activating DNAzymes. These DNAzymes identify and cleave the marked Egr-1 gene to achieve the gene-silencing function (Figure 5B). The Apt/Dz nanostructure shows the advantages of targeted delivery, better stability, nontoxicity, and reduces side effects which are essential in using DNAzymes as therapeutic drug (Li et al., 2020).

### Gels and Switchable Networks

For drug delivery and controlled targeted release of the drug, much attention has been drawn toward developing switchable microgels, which can quickly enclose pharmaceutical compounds and deliver them when triggered by a particular stimuli (Galaev and Mattiasson, 1999; Ahn et al., 2002). Yurke et al. developed a convertible gel system based on DNA and is formed by the copolymerization of acrylamide with DNA strands. Chemical modification of DNA strands was done with reactive group acrydite (Lin et al., 2004). The gel formed can easily be switched to the gel state from the fluid state when there is crosslinking between the acrylamide DNA strand and the complementary DNA linker strand. The amount of cross-linker strand decides the mechanical properties of the gel. If the cross-linker strands are removed using the removal strand through the “strand displacement” method, the gel will be converted back to the fluid state. Later they also show that fluorescent nanoparticles can easily be trapped in the DNA-polyacrilamide gel and can be released upon addition of suitable effector DNA. Mi and coworkers in some what similar way used crosslinking DNA strands. containing sequences of thrombin binding aptamers. These crosslinking DNA are used to load thrombin on to the gel. The resolution of the gel depend upon the amount of thrombin loaded on to the gel. In response to any chemical stimuli this gel can release drug-carriers to the target site.

**FIGURE 6:**
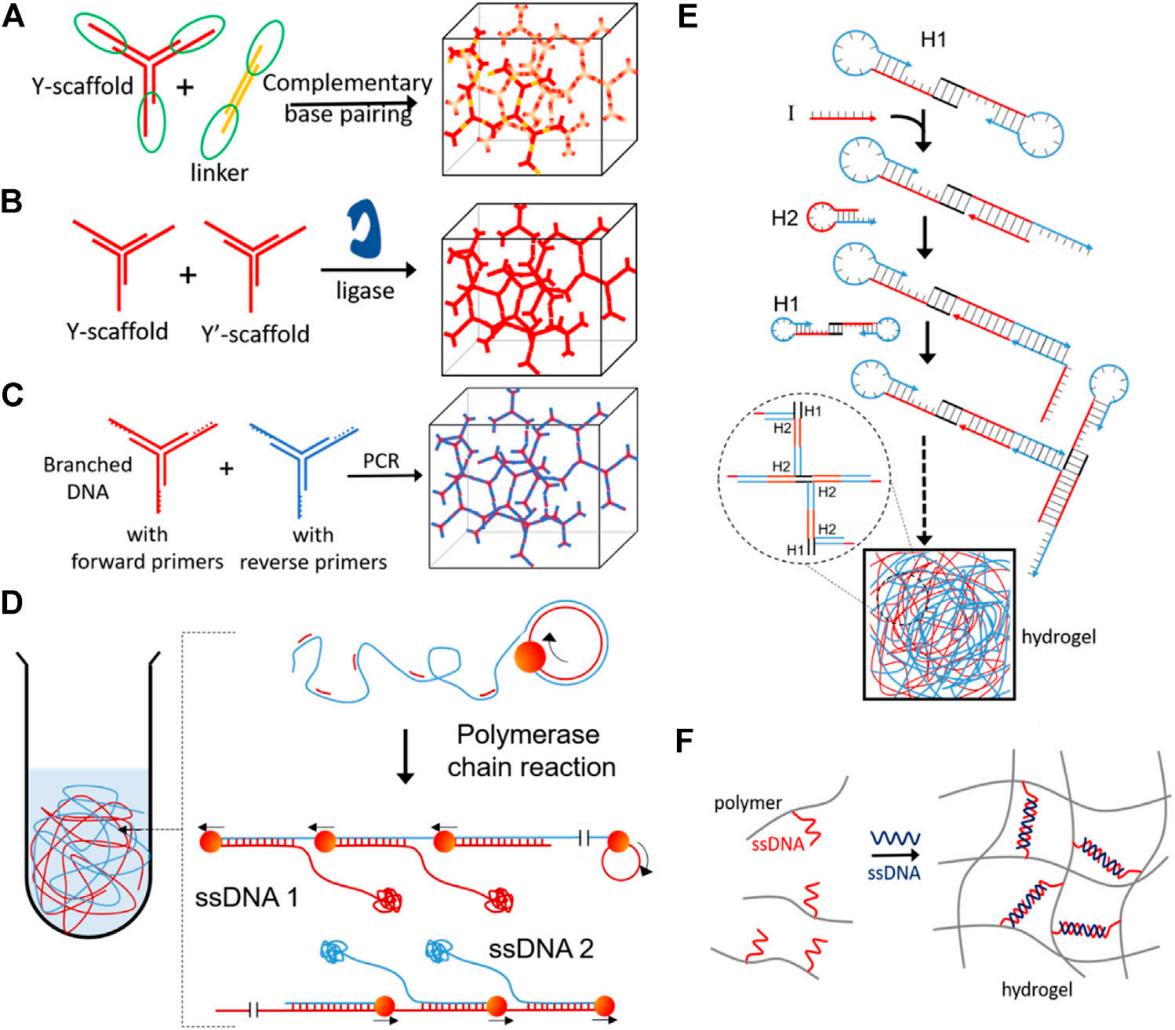
DNA based hydrogels formation by **(A)** self-assembly of complimentary base pairing (Xing et al., 2011), **(B)** enzymatic ligation (Um et al., 2006), **(C)** polymerase chain reaction (Hartman et al., 2013), **(D)** DNA synthesized *in situ via* rolling circle amplification (RCA) having a large amount of physical entanglement (Lee et al., 2012b), **(E)** hybridization chain reaction (Wang et al., 2017b) and **(F)** DNA base pairing induced gelation of acrylamide (Nagahara and Matsuda, 1996). Reproduced with permission from ref Morya et al. (2020). Copyright 2020, American Chemical Society (ACS).

Switchable DNA hydrogels can also be made exclusively from DNA. The simple and easiest way to form DNA hydrogels is by polymerizing branched DNA motifs like a three-way junction or four-way junction, developing through self-assembly of DNA hydrogels (Nagahara and Matsuda, 1996; Li et al., 2016b; Shahbazi et al., 2018; Morya et al., 2020) (Figure 6A). Different methods are being used to enhance the assembly rate of DNA hydrogel formation, like the use of ligating enzymes (Figure 6B) (Um et al., 2006), polymerase chain reaction (Figure 6C) (Hartman et al., 2013), rolling circle amplification (Figure 6D) (Lee et al., 2012b), hybridization chain reaction (Figure 6E) (Wang et al., 2017b). DNA can also be used as a crosslinking agent between polymer chains to form responsive hydrogels (Figure 6F) (Nagahara and Matsuda, 1996). DNA hydrogels become an intelligent platform for various biomedical applications because of their stimuli-responsive functioning and nontoxicity, and good biocompatibility (Kahn et al., 2017; Wang et al., 2017d). The size and shape of the colloidally stable DNA hydrogels can be changed from few nanometers to bulk gels ranging up to micrometers (Thelu et al., 2017). The DNA gels having a high surface-to-volume ratio at the nano and microscopic levels make them readily available to target diseased tissue or cells (Mou et al., 2017).

DNA based responsive hydrogels have been explored in many areas, majorly in sensing, capturing, and controlled release (Xiong et al., 2013). Different DNA based hydrogels responsive to various stimuli like pH, ions, biomolecules, enzymes, cDNA have been developed to apply in sensing and biomedical applications (Morya et al., 2020). Sensing of Cu^+2^ has been demonstrated by using Cu^+2^ DNAzyme sequence as the cross-linker for polymer matrix. In presence of Cu^+2^, the cross-linkers break into fragments and release the gold nanoparticles embedded in the hydrogel matrix, as a readout (Lin et al., 2011). In a similar approach, Dave et al. used Hg^+2^ specific aptamer as a cross-linker and detects Hg^+2^ in water samples (Dave et al., 2010). Therapeutic gene delivery is an emerging field in biomedical engineering, enzyme responsive DNA nanogels decorated with aptamer utilized for targeted delivery and release of a therapeutic gene inside cells (Li et al., 2015). Specific recognition and recovery of small molecules have also been realized using DNA based hydrogel. He at el. captured and recovered adenosine-5′-triphosphate (ATP) molecules from a mixture of molecules using an aptamer cross-linked reversible DNA based hydrogel (He et al., 2010). The highly modular construction and versatile components make the DNA based hydrogels suitable for a plethora of applications.

### Future Directions and Perspectives

DNA has been beyond demonstrated to be an excellent scaffold for realizing artificial molecular machines. Because of the predictable nature of these sequence-dependent structure formations by the DNA molecules, programmable supramolecular structure self-assembly can be created that switched into different conformations when given particular stimuli. The essential advantage in using nucleic acid scaffolds is that they are formed by other units and can easily couple to various functional materials present on a single structure. Both chemical technology and biochemistry are being used to synthesize these devices in considerable quantity. Dynamic DNA nanotechnology is one of the crucial developing sub-branch of DNA nanotechnology. The achievements and success achieved are based on DNA nanostructures, including structural programmability, synthetic accuracy, ease of chemical modification, nontoxicity, compatibility with other functional nucleic acids, and pre-well-understood knowledge of different DNA-based structures. Given the developments in DNA structural devices as well as new chemistries available for its coupling to an arena of responsive biomolecules, DNA nanodevices are poized to metamorphose soon into next generation stimuli-responsive materials with applications in broad spectrum areas like biosensing, targeted delivery, immunotherapy, chemotherapy to mention the few.

Despite so many advantages, there are still so many challenges to deal, in the field of stimuli-responsive DNA nanodevices. In most cases, the devices’ response time is comparatively slow because of accompanying structural changes, which take minutes to hours to finish. The overall efficiency and reversibility of the process are also decreased because of multiple steps. Also, the wastes such as salt and hybridized DNA accumulated after any chemical trigger reaction may affect the device’s performance. Significant potential in the construction of sophisticated responsive DNA nanodevices has been shown by DNA origami. However, protection against enzymatic degradations and structural disassembly is required. It is still difficult to predict the structural changes in DNA nanodevices once used for *in vivo* applications. Diverse non-biological stimuli like magnetic, electrical, and optical controlled stimuli-responsive DNA structures are chosen for a cleaner and non-invasive application method in the coming years. For example, i-motif’s switching by electrolytically generating bases and acids has already been achieved by Liu et al. (Del Grosso et al., 2015). Simmel. et al. have developed DNA-origami based nanomechanical setup combined with an electrically manipulated robotic arm under synchronously alternated, quadrupolar electric fields (Kopperger et al., 2018). All these physical sources are especially welcome as they are a non-invasive method triggering mechanism. Responsiveness to light is much easier to deal with, as compared to electric responsiveness. Near-infrared light is better to use as compared to ultraviolet light due to good biosafety and penetration depth. Several triggering methods for highly integrated systems can be used in the future to perform complicated tasks.

Apart from DNA bases, which occur naturally, non-natural nucleotide analogs are good postulants for excellent stimuli control and biomedical application. Floxuridine-incorporated DNA self-assemblies prepared by Zhang.et al. (Chen et al., 2018) shows superior anti-cancer efficacy. Considering the exceptional properties of DNA-based stimuli-responsive systems and rapidly emerging research in this field, we could see a big future for DNA devices in direct biomedical applications like recent covid19 biosensing.
